# Analysis of Efficiency of Thermoelectric Personal Cooling System Based on Utility Tests

**DOI:** 10.3390/ma15031115

**Published:** 2022-01-31

**Authors:** Anna Dąbrowska, Monika Kobus, Łukasz Starzak, Bartosz Pękosławski

**Affiliations:** 1 Department of Personal Protective Equipment, Central Institute for Labour Protection-National Research Institute, Wierzbowa 48, 90-133 Lodz, Poland; monikakobus98@gmail.com; 2 Department of Microelectronics and Computer Science, Lodz University of Technology, Wólczańska 221/223 B18, 90-924 Lodz, Poland; lukasz.starzak@p.lodz.pl (Ł.S.); bartosz.pekoslawski@p.lodz.pl (B.P.)

**Keywords:** thermoelectric effect, Peltier modules, thermoelectric cooler, personal cooling, smart clothing

## Abstract

Thermoelectric modules can find practical application in clothing with a cooling function. A personal cooling system using Peltier modules integrated with clothing was developed and tested with the participation of a person. A dedicated electronic controller was designed that enabled the power or temperature to be controlled and recorded. In the research, the influence of heat sinks and the method of controlling the operation of the module on the cooling efficiency was assessed. The research was aimed at selecting the operating mode of the controller and choosing the arrangement of modules comparing cooling efficiency. The research showed that by selection of appropriate controlling mode, the electric power used can be reduced while keeping the cooling efficiency at the same level. The location of Peltier modules in places where they can tightly adhere to the body increases their performance.

## 1. Introduction

In order to avoid the risks caused by the long-term exposure of workers to a hot environment, it is necessary to eliminate or limit the accumulation of heat in the body. However, there are many workplaces where, due to technological processes or large spaces, it is not possible to use air-conditioning systems. Due to the lack of other possibilities of cooling the employee’s body during work, the solution to this problem may be personal cooling systems. Therefore, work is underway to design such systems that are effective in drawing the excess heat generated by the human body during work.

The cooling process associated with the Peltier effect has been practically implemented in thermoelectric coolers (TECs), also called Peltier modules [1]. These consist of a number of alternating pairs of n-type and p-type semiconductors, connected thermally in parallel and electrically in series, sandwiched between two plates that conduct heat and are electrical insulators [2]. There are reports in the scientific literature where thermocouples have found practical application in clothing with a cooling function. An example of such use is the jacket developed by Lavanya et al. [3]. A very interesting solution is also a jacket that enables both cooling and heating of the user’s body depending on their needs, developed by Poikayil et al. [4]. It is a battery-powered system with a built-in temperature sensor that measures the internal temperature of the vest. The cooling and heating effects are ensured by Peltier modules with fans, integrated with the jacket. Clothing with a cooling function using highly efficient Ecoflex flexible modules was developed by Hong et al. [5]. The authors proposed a vest and a headband to be used directly on the body.

Due to the significant miniaturization and technological progress observed in recent years in the field of wearable electronics, the use of flexible Peltier modules for the purpose concerned seems to be a promising direction. Their undoubted advantages are small dimensions, low weight, high durability and resistance to mechanical damage, as well as low production costs [6]. The conducted research shows that cooling systems based on the thermoelectric phenomenon are able to stabilize the microclimate temperature under clothing during physical activity, which confirms the effectiveness of their operation [7]. Moreover, thanks to Peltier modules being principally semiconductor devices, the reliance on electronic solutions allows the temperature in the undergarment microclimate to be easily monitored and controlled to ensure thermal comfort [4].

## 2. Materials and Methods

### 2.1. Tested Object

**Figure 1:**
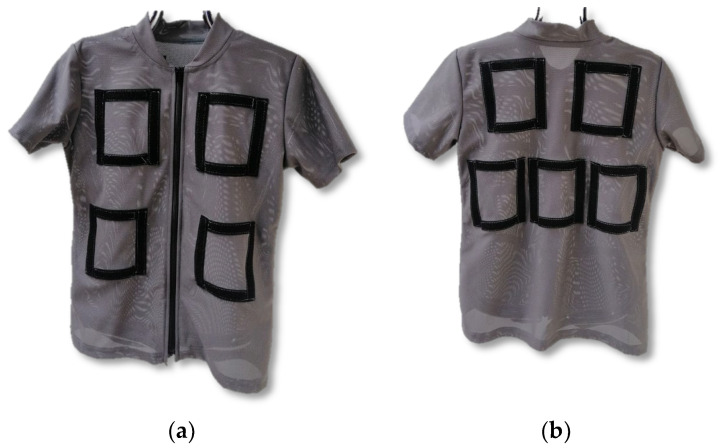
Clothing dedicated to use with the developed personal cooling system: (**a**) front; (**b**) back.

**Figure 2:**
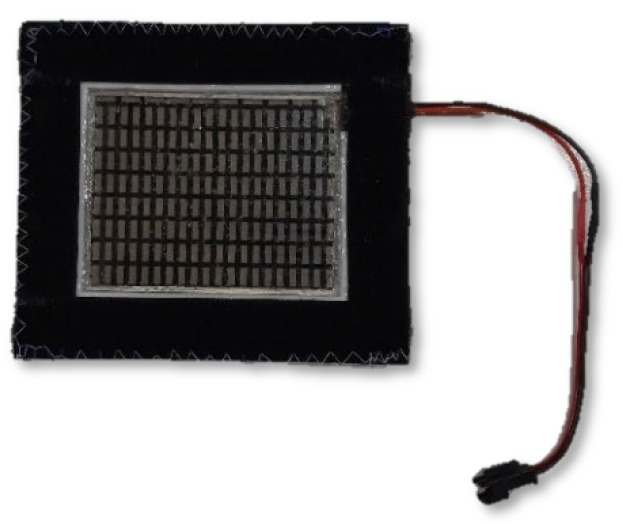
Flexible Peltier module TEGway FTE1-01 in a holder that can be placed on the blouse.

**Figure 3:**
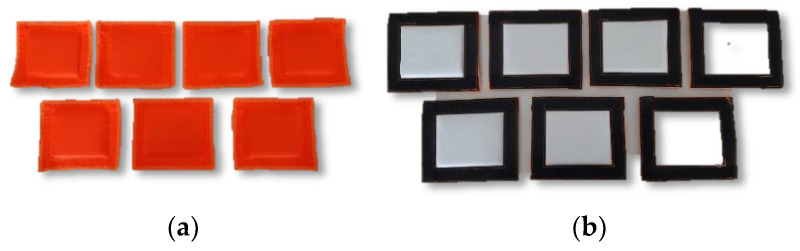
Developed heat sinks: (**a**) top view; (**b**) bottom view.

**Figure 4:**
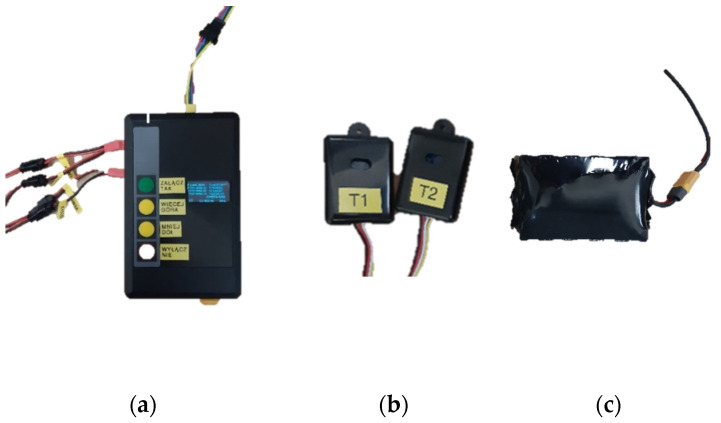
Components of the cooling system: (**a**) controller with visible sensor, battery pack and thermoelectric module cables; (**b**) temperature and humidity sensors; (**c**) battery pack.

As a part of this research, a model of an active personal cooling system based on the thermoelectric phenomenon was developed and investigated. For the purpose of performing laboratory tests of the developed personal cooling system, special clothing was designed in order to ensure the possibility of various arrangements of Peltier modules located on the human body. Therefore, laboratory tests were performed on an active personal cooling system with Peltier modules integrated with clothing, that specifically included:

- clothing with a mounting system (Velcro holders) in nine locations intended for Peltier modules (Figure 1);
- seven Peltier modules (Figure 2) and heat sinks for integration with clothing (Figure 3) with Velcro holders;
- a dedicated electronic controller enabled the electric power of the Peltier modules or the temperature to be controlled, monitored and recorded (Figure 4).

The clothing was made of Coolmax openwork knitted fabric and had a form of a T-shirt (Figure 1) on which seven Peltier modules can be placed in nine different locations by means of Velcro tapes.

The system was designed to perform the cooling function by means of the aforementioned modules and could supply a maximum of seven such modules (Figure 2). Peltier modules were adapted so that they could be placed on clothing by closing them in silicone frames on which Velcro tape was placed on both sides.

On each Peltier module, one heat sink (Figure 3) was placed also by means of the Velcro holder. The heat sinks were made using the SAF (Super-Absorbent Fabric) Fabric 2644 superabsorbent non-woven fabric by Technical Absorbents which was inserted between the S1TA55 fabric (top layer) and the F01 knit (backsheet). Thanks to the use of developed heat sinks made of a superabsorbent non-woven fabric able to absorb a high amount of water and prevent its leakage, heat generated by a hot side of the Peltier module was dissipated through conduction and evaporation. Therefore, providing fabrics with a low thermal and water vapor resistance was essential for effective operation of the heat sinks.

The developed personal cooling system consists of an electronic controller (Figure 4a), two temperature and humidity sensors (Figure 4b) and a lithium-ion battery pack (Figure 4c). The sensors enable temperature to be measured in the range from −40 °C to 60 °C. The controller enables various configurations of the cooling system to be created. In order to regulate the power of the Peltier modules, the controller generates constant voltages of appropriate values at the outputs of channels 1 to 3 and monitors the respective currents. This allows a different electric power to be supplied in each channel and, consequently, a different cooling power to be obtained, understood as the heat transferred to the environment per unit time. The controller allows the user to control the electric power supplying the modules in two ways. The first one is manual control where temperature sensors are not used and the user sets the value of the module supply power. The controller adjusts the output voltage while measuring the output current to obtain the desired power. A second control method is applied in the automatic mode, which becomes available after connecting at least one temperature and humidity sensor. In this case, the user sets the value of temperature, while the controller automatically determines the module supply power to obtain the desired temperature using temperature values collected from the sensors. If two sensors are connected the controller’s operation is based on the average of the temperature values measured by these sensors. The power setting is identical for all the channels, regardless of the control mode. The controller can be powered from any DC power source with an output current of at least 4 A and a voltage in the range from 12.5 V to 21.0 V. Its maximum output power is 41.3 W. The structure and operating principle of the controller is described in detail in Section 2.2.

**Figure 5:**
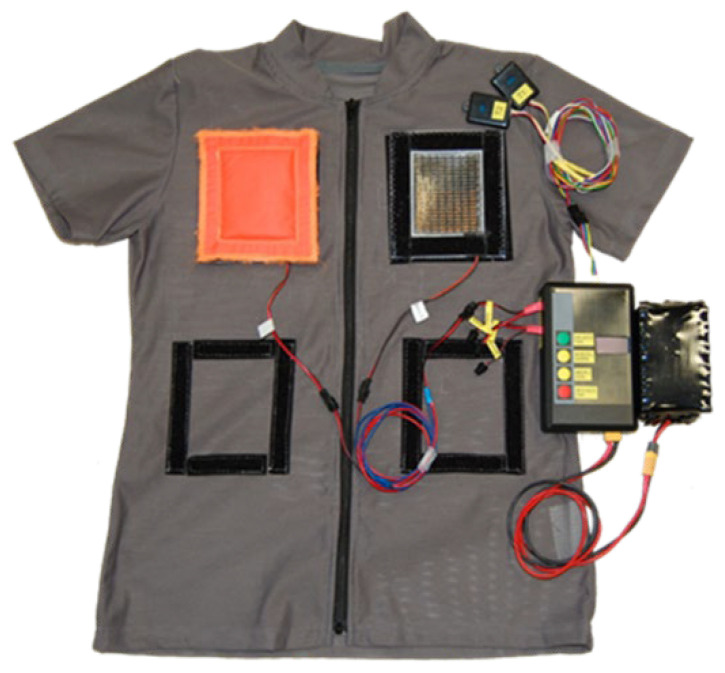
A view of the tested object after integration.

A complete tested object is presented in Figure 5. For better visualization, in the photo only two Peltier module were integrated and only one was covered by the heat sink; however, all tests presented in the paper included the use of seven modules and seven heat sinks covering each Peltier module.

### 2.2. Structure and Operating Principle of the Dedicated Electronic Controller

**Figure 6:**
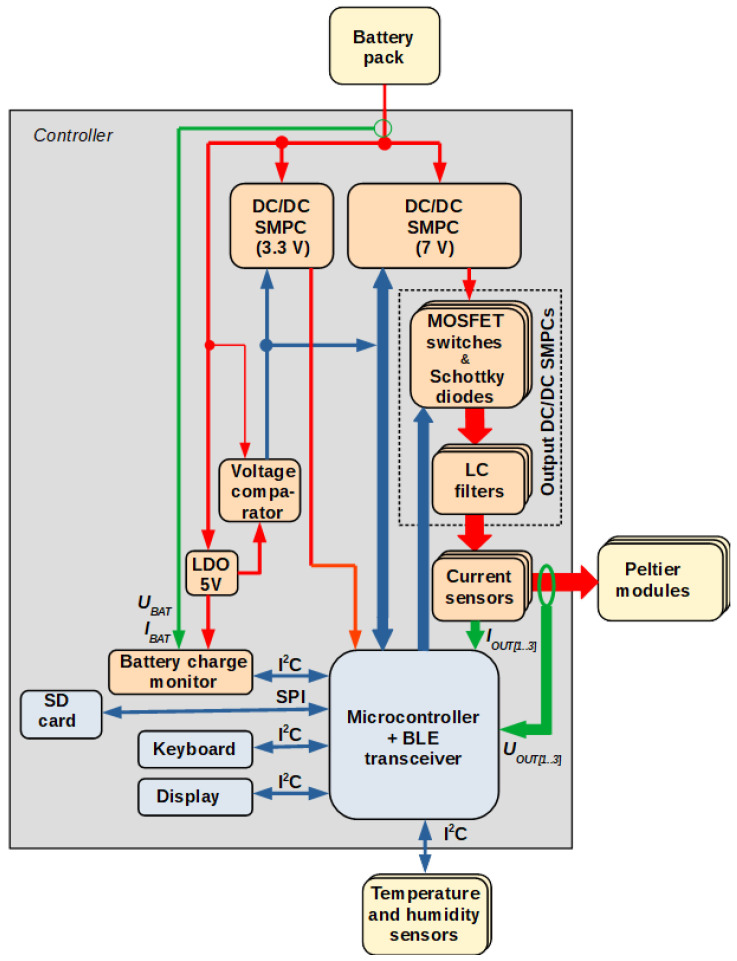
Block diagram of the cooling system controller (digital signals: blue, analog signals: green, power paths: red).

The essential part of the tested object was the dedicated electronic controller. The block diagram of the controller is shown in Figure 6. Its main components are:

- an auxiliary DC/DC switched-mode power converter (SMPC) with an integrated analog controller, to supply the control electronics;
- a main DC/DC SMPC with an integrated analog controller, to derive power for the TEC;
- three digitally controlled output DC/DC SMPCs (one per output channel), to supply the Peltier modules in each channel;
- current sensors (one per channel) in the form of shunt resistors with dedicated voltage amplifiers, to measure channel output currents;
- a battery charge monitor supplied from a separate low-drop-out (LDO) linear voltage regulator;
- a voltage comparator turning off the converters when the battery voltage becomes to low, thus protecting the battery from excessive discharge;
- a microcontroller with the Bluetooth Low Energy (BLE) radio interface;
- a four-button keyboard;
- an alphanumeric organic light emitting diode (OLED) display;
- a micro secure digital (SD) card interface for configuration and data storage.

The auxiliary and the main power converters regulate their output voltages to the necessary levels: 3.3 V for the control electronics and 7 V for the TEC, regardless of the battery voltage or output currents. The output power converters employ the buck topology, each of them being composed of a MOSFET (metal-oxide semiconductor field-effect transistor) switch, a Schottky diode and an LC filter; they are driven by pulse width-modulated (PWM) signals generated by the microcontroller. The filters ensure constant output voltages with reduced ripple.

The microcontroller measures the channel voltages and currents using a built-in analog-to-digital converter (ADC). It also communicates with the external temperature and humidity sensors, the battery charge monitor, the keyboard and the display via the inter-integrated circuit (I2C) interface and with the SD card via the serial peripheral interface (SPI) bus. The BLE interface can be applied in the future for remote control, e.g., with a smartphone or smartwatch.

The power controller has been implemented as a digital proportional one with feedforward, where the output quantity is the duty cycle of the respective output converter switch. It is run with a constant period of 50 ms. The ideal voltage equation of the buck topology is [8]:

U_o_ = D U_i_, (1)

where D is the duty cycle, U_i_ is the input voltage and U_o_ is the output voltage. The input voltage value is constant and known (7 V) as it is derived from the main power converter. The output voltage determines the output power according to the Joule’s law:

P_o_ = U_o_^2^ G_pm_, (2)

where P_o_ is the output power and G_pm_ is the equivalent conductance of the Peltier modules in the given channel. By combining Equations (1) and (2), the formula for the feedforward term of the controller D_ff_ is obtained:

D_ff_ = (P_o(aim)_ G_pm_)^1/2^/U_i_, (3)

where P_o(aim)_ is the output power aimed at. On the other hand, the proportional feedback term D_p_ is calculated as:

D_p_ = −K_p_ (P_o(meas)_ − P_o(aim)_), (4)

where K_p_ is the proportional term coefficient and P_o(meas)_ is the output power measured, obtained by multiplication of measured output voltage U_o(meas)_ and current I_o(meas)_:

P_o(meas)_ = U_o(meas)_ I_o(meas)_, (5)

The ultimate output of the power controller is the sum of the feedback and feedforward terms:

D = D_ff_ + D_p_, (6)

The output power aim is related to, but not identical with, the output power set by the user. This is to avoid the sudden feeling of cold or warmth when the cooling power is increased or decreased, including at start-up or turn-off. The aim is, therefore, updated up or down to the set value in steps every 400 ms.

In the automatic mode, the power controller still operates. However, the power setting is no longer imposed by the user, but instead it is determined by a higher-level temperature controller. As temperature variations are relatively slow, the latter is run approximately every 500 ms. Temperature regulation with Peltier modules is frequently achieved using full proportional-integral-derivative (PID) controllers, requiring the parameters of the plant to be first identified in order to determine the optimum term coefficients [9]. More recently, a fractional-order PID controller was proposed [10]; although it improves performance, its implementation and optimization is more demanding. A PI or P + I offers a simpler solution, but still requires the transfer function of the plant to be known and a transfer function zero to be cancelled out [11].

In the considered case, plant parameters vary between users as well as in time. Therefore, it was decided to implement a simpler, proportional-derivative (PD) controller, which has the advantages of a simple digital formula and high stability. At the same time, the presence of the derivative term enables a power boost to be produced when the set temperature or the measured temperature changes abruptly. The implemented controller’s equation is:

P_o(set)_ = K_pT_ T_e[i]_ + K_dT_ (T_e[i]_ − T_e[i−1]_)/(t_[i]_ − t_[i−1]_), (7)

where K_pT_ and K_dT_ are the proportional and derivative term coefficients, respectively, t is the time associated with measured temperature samples, i is the current temperature sample number, and T_e_ is the temperature error, defined as:

T_e_ = T_meas_ − T_set_, (8)

where T_meas_ is the temperature measured and T_set_ is the desired temperature set by the user.

Irrespective of the current operating mode of the controller, as long as the outputs remain active, measurement data are periodically stored on the SD card. They include: time, battery voltage and current, set and measured temperatures, measured relative humidity, power setting as well as each output channel voltage and current.

### 2.3. Testing Methodology

#### 2.3.1. Research Conditions

The research was carried out with the participation of a human in a research and demonstration laboratory which enables constant environmental conditions to be maintained, including ambient temperature, relative humidity and air velocity. The tests were carried out in an environment with a temperature varying from 25 °C to 35 °C in steps of 5 °C, a constant relative humidity of 65% and a constant air velocity of 0 m/s.

#### 2.3.2. Measured Parameters

During experiments, both thermal and electrical parameters were measured. In terms of thermal parameters, the focus was primarily on the skin temperature of the user. The temperature was measured using AC1913-A sensors from Rotronic. These are Class A (four-wire) Pt100 temperature sensors that measure temperatures in the range from −50 °C to 200 °C. The measured temperature was recorded using the HygroLog HL-NT2-D humidity and temperature recorder and the HL-DS-U2 docking station, which allows the connection of four AC1913-A sensors simultaneously.

In terms of electrical parameters, the voltages and currents of the Peltier module channels as well as the electrical power of a single module for each of these channels were measured by the dedicated controller and recorded on an SD card.

#### 2.3.3. Research Procedure

In order to standardize the conditions for this study, the clothing, the cooling system, Peltier modules and the heat sinks were acclimated in a separate air-conditioned room at a temperature of 23 °C for 1 h before the experiment. The same applied to the participant. Conditions in the laboratory stabilized for 2 h prior to the start of the experiment.

Heat sinks were prepared before starting the experiment as well. This was done by soaking them in water at room temperature of approximately 23 °C until their weight reached between 80 g and 90 g. At the same time, the clothing was prepared for the test by placing the Peltier modules on it in designated places (depending on the stage of the study), which was then put on by the participant. The controller of the cooling system together with the battery pack were inserted into a pelvic kidney placed in the waist of the test participant. After soaking the heat sinks, they were attached to the Peltier modules on the clothing using Velcro tapes. As good adhesion of Peltier modules to the skin is crucial for an efficient operation of the cooling system, the adherence of the blouse to the participant’s figure was improved by fastening any excess material of the clothing.

After preparation and acclimatization, the participant entered the laboratory. The tests were performed with a use of the Zebris FDM-THM-M-3i treadmill by Zebris Medical GmbH according to the following six-stage procedure:

- Activity I—walk at a speed of 3 km/h.
- Break.
- Activity II—walk at a speed of 5 km/h.
- Break.
- Activity III—walk at a speed of 5 km/h with a 10% inclination of the treadmill.
- Break.

#### 2.3.4. Study Variants

**Figure 7:**
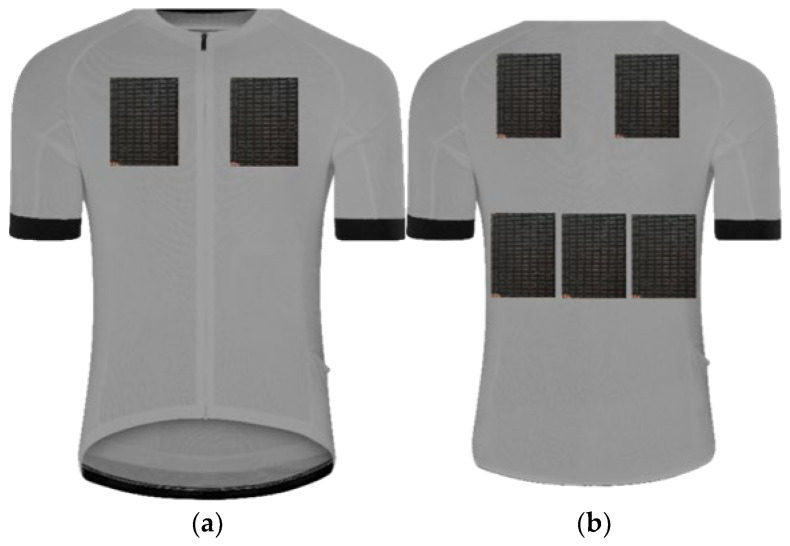
Peltier module arrangement on the blouse, arrangement I: (**a**) front side; (**b**) back side.

**Figure 8:**
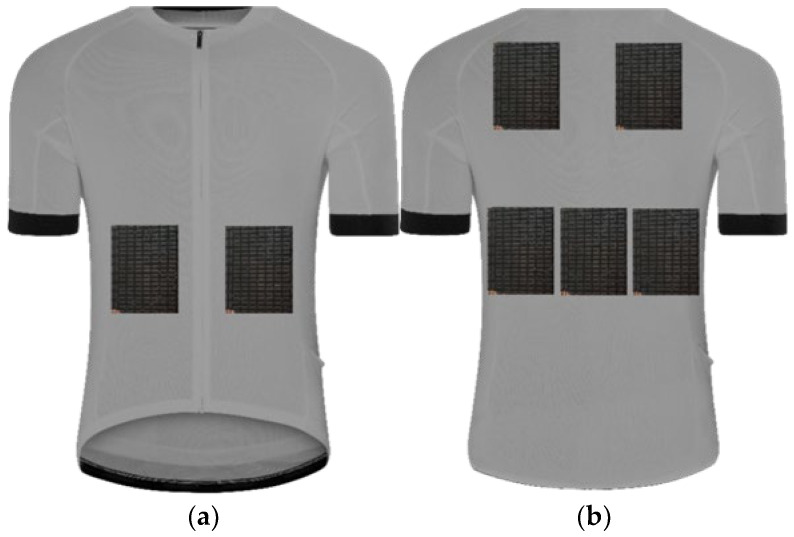
Peltier module arrangement on the blouse, arrangement II: (**a**) front side; (**b**) back side.

**Figure 9:**
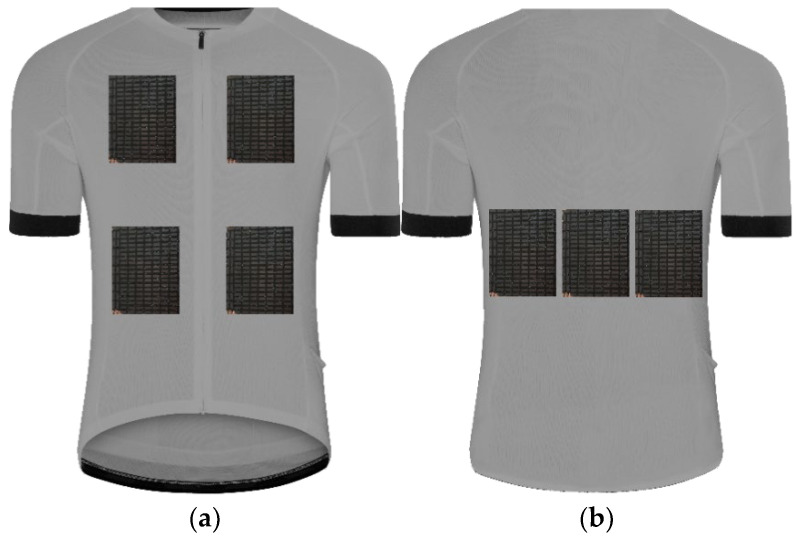
Peltier module arrangement on the blouse, arrangement III; (**a**) front side; (**b**) back side.

The laboratory tests were divided into two successive stages presented in Table 1. The consecutive stages were aimed at selecting the cooling mode (Stage I) and choosing the arrangement of Peltier modules (Stage II) in terms of ensuring the highest cooling efficiency with limitations of the electric energy consumption. At Stage II, three different arrangements were considered:

- 2 modules on the chest plus 5 modules on the back (Figure 7),
- 2 modules on the abdomen plus 5 modules on the back (Figure 8),
- 2 modules on the chest plus 2 modules on the abdomen plus 3 modules on the back (Figure 9).

## 3. Results

### 3.1. Control Mode

**Figure 10:**
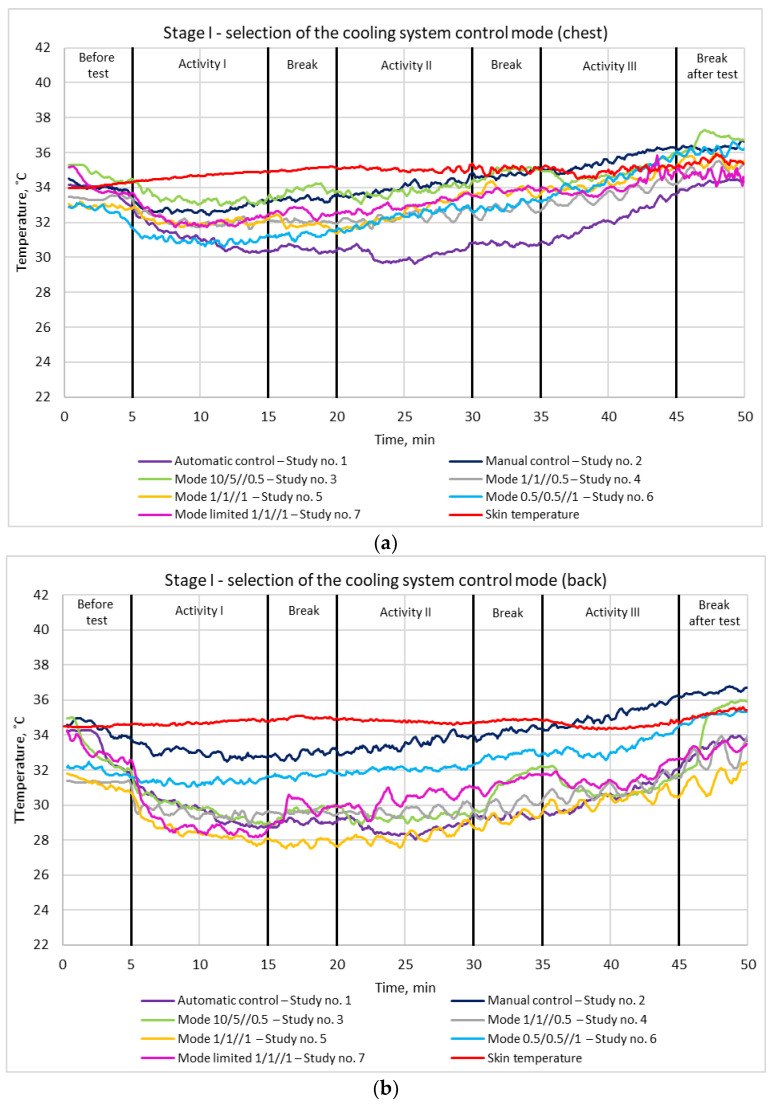
Variation of skin temperature under the Peltier modules and next to them (Stage I of the study): (**a**) chest; (**b**) back. Study number complies with Table 1.

**Figure 11:**
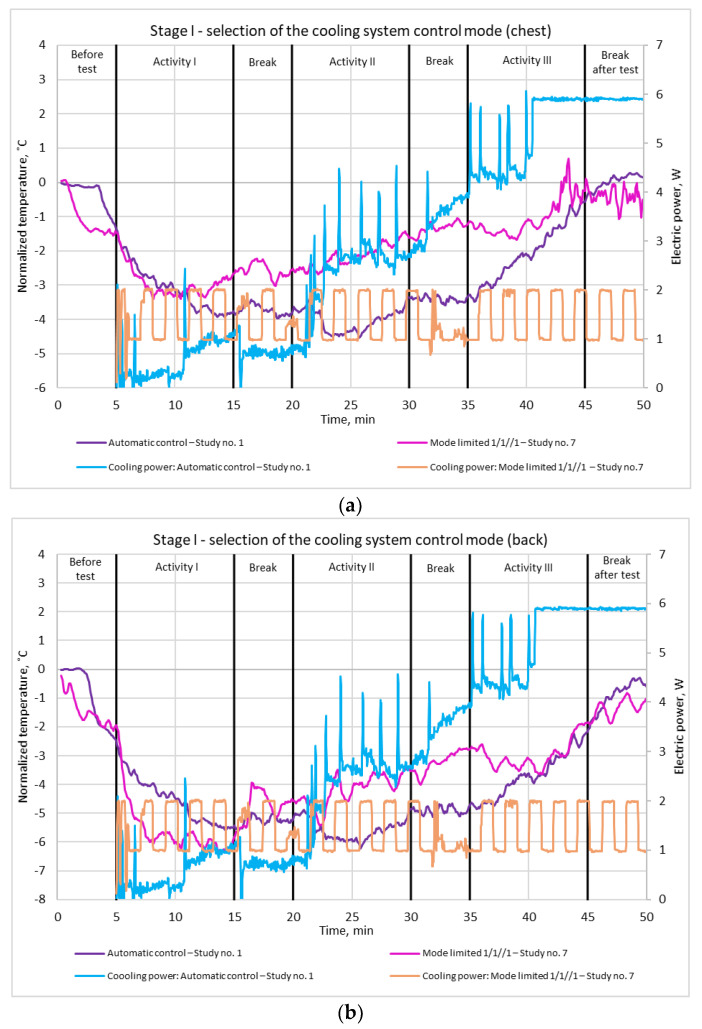
Variation of normalized skin temperature under the Peltier modules for the two selected control modes, together with average supply power per Peltier module: (**a**) front; (**b**) back. Study number complies with Table 1.

Seven control modes have been tested at Stage I, as indicated in Table 1. Figure 10 and Figure 11 show the results of skin temperature measurements under the Peltier modules and next to them, for the different modes, either on the chest (Figure 10a) or on the shoulder blades (Figure 10b).

For all the control modes, the measured temperature under the modules on the chest was higher than that measured on the shoulder blades. While analyzing the temperature on the chest (Figure 10), it can be concluded that the lowest values were obtained for the automatic mode, reaching down approximately 30 °C during activity II. On the other hand, for the manual mode, the 10/5//0.5 mode and the 0.5/0.5//1 mode, the temperature under the modules at the end of the test exceeded the temperature of the skin next to the modules, which testifies to an effect opposite to that intended (cooling). For this reason, these modes cannot be applied in the system. For the limited mode 1/1//1 the lowest temperature, of approximately 32 °C, was measured during activity I, and at the end of the test, the temperature was approximately 1 °C lower than the temperature of the skin next to the modules. A similar result at the end of the test was also obtained for the automatic mode. The observed increase in skin temperature under the Peltier modules was caused by insufficient heat removal by the heat sinks from the hot sides of the modules.

As for the temperatures measured on the shoulder blades (Figure 11), the lowest was achieved for the 1/1//1 mode and amounted to approximately 28 °C after activity I. For that mode, as well as for the automatic mode, the 1/1//0.5 mode and the limited mode 1/1//1, the temperature values at the end of the tests were similar, between 32 °C and 34 °C.

Based on the above observations, the automatic mode and the limited mode 1/1//1 were selected for further consideration. Variations of the normalized (i.e., referred to as the ambient) skin temperature under the modules are presented for these two modes in Figure 11a,b, together with the average supply power per Peltier module.

It can be concluded that a stronger cooling effect was initially obtained for the automatic mode: a decrease in skin temperature of about 4.5 °C was observed on the chest and of 6 °C on the back in the 25th minute of the experiment. This resulted from a much higher supply power being applied to the modules than the power limit imposed in the other mode: from the 23rd minute, the supply power per module exceeded 2 W and increased up to about the 40th minute, when it reached its maximum value (limited by the controller’s ratings) of 6 W. However, from the 26th minute onward, the skin temperature under the modules on both the chest and the shoulder blades began to rise. This was due to the fact that with such a high supply power, the power dissipated in the Peltier modules as a result of their operation was much higher than at lower power values, which increased the total heat to be removed from the hot sides of the modules above what could be handled by the heat sinks.

Based on the above conclusion, the supply power was limited to 2 W in the complex mode, where automatic operation intervals alternate with standby operation with supply power decreased to 1 W This resulted in a temperature decrease of about 3 °C on the chest and of 6 °C on the back in the 10th minute. Additionally, it should be noted that for this control mode, the temperature at the end of the test did not exceed the initial value while total power consumption was lower than with the automatic mode.

When selecting the cooling system control mode, the subjective feelings of the test participant were also taken into account. Thanks to the introduction of standby intervals, the cooling effect was felt much longer and more often than with automatic control. This was because immediately after each standby interval, skin temperature was relatively high, so the controller, operating in its automatic mode, increased the module supply power to the maximum.

On this basis, the control mode of 1 min/1 min (auto/1 W) with a 2 W power limitation (Mode limited 1/1//1) was selected for further stages of the study.

### 3.2. Peltier Module Arrangement

**Figure 12:**
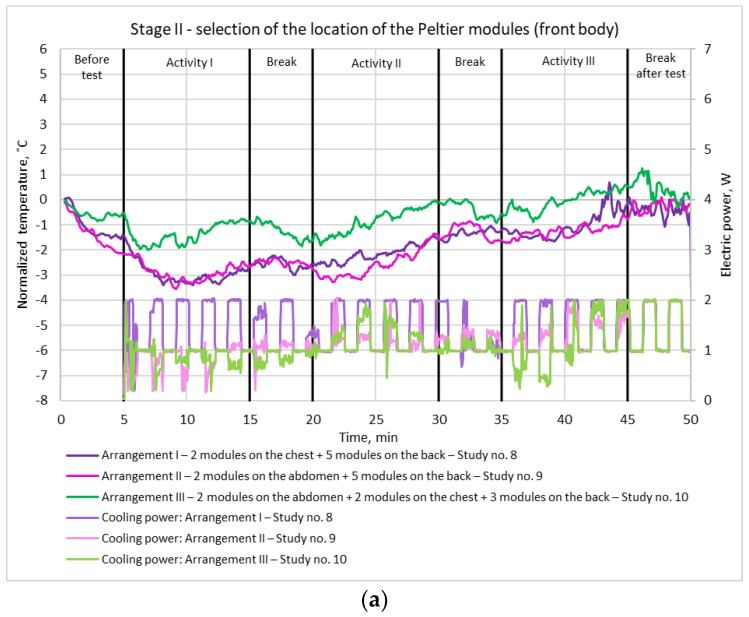

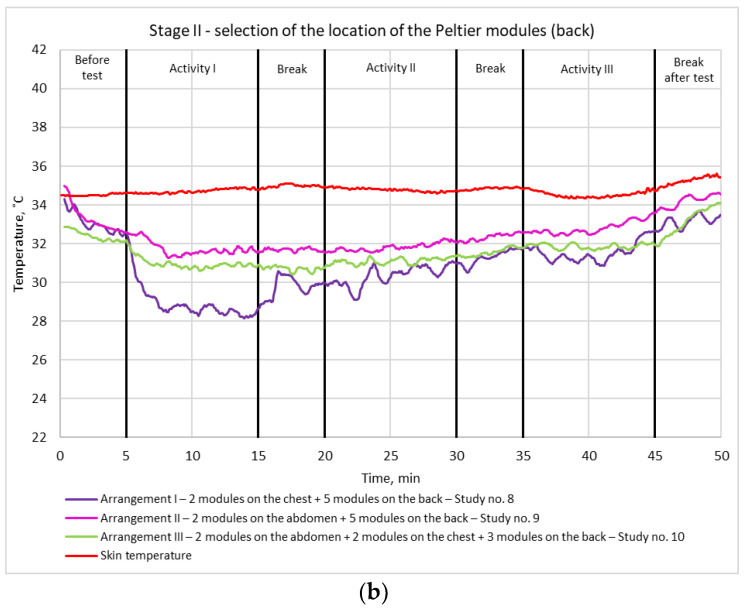
Variation of temperature under the Peltier modules and next to them for three module arrangements: (**a**) normalized skin temperature at the front; (**b**) skin temperature at the back. Study number complies with Table 1.

In order to determine the optimal locations of thermoelectric modules on the blouse, three arrangements of module arrangement were tested. Figure 12a shows variation in the normalized skin temperature under the modules on the front of the body together with average supply power per Peltier module, and Figure 12b shows variations in skin temperature under the modules on the back, for the particular arrangements.

It can be seen that the lowest temperatures on the front of the body (Figure 12a) were generally recorded for arrangement II, where the Peltier modules were placed on the abdomen. Temperatures for arrangement I were similar, although generally higher, while for arrangement III, where the modules were located both on the abdomen and on the chest, they were always much higher than for the other arrangements. The temperature on the shoulder blade for arrangement I throughout this entire stage of the study was very similar to the temperature on the abdomen for arrangement III.

The temperature measured on the back (Figure 12b) was lower for arrangement I than for arrangement II. However, although module locations on the back were the same in both cases, the temperature was measured for a module placed on the shoulder blade for arrangement I, while for arrangement II it was measured for a module located at the lower back.

In general, similar cooling profiles were obtained for arrangements I and II throughout the experiment. However, module power consumption was definitely higher for arrangement I than for arrangement II, staying at the limit of 2 W for almost the entire duration of the experiment. It follows that the application of arrangement I is not economically viable, since a similar level of cooling can be obtained by using less power with arrangement II. It should also be noted that for arrangement III, where modules were placed on the abdomen, the temperature was on average about 2 °C higher than in arrangement I. This indicates a strong influence of the heat sinks on the resulting cooling effect.

Based on the analysis, arrangement II was selected for further stages of the study.

## 4. Discussion

In terms of skin temperature under the modules, the best results were achieved when the complex control mode was applied, consisting in alternating between automatic mode and standby intervals. Limiting of the supply power in the automatic phase is necessary because, otherwise, heat dissipation in the module during its operation may become higher than what could be effectively removed by the heat sink, leading to the reversal of the thermal effect (skin heating instead of cooling). On the other hand, the standby supply power should be low, but not zero, because the latter results in thermal discomfort being felt by the user. Nevertheless, the strongest initial cooling effect was obtained for continuous automatic mode. This proves that the digital power and temperature controller has been designed correctly for the considered purpose. The results obtained from the tests with a human participation are in accordance with laboratory tests performed on the Peltier modules with the use of an apparatus called a ‘skin model’ [12] which indicated that it is necessary to limit the supply power in order to ensure effective operation of the modules. Moreover, a possibility to provide a cooling temperature of up to 5 °C by means of a system based on the thermoelectric modules was also confirmed by Mitsik et al. [13] and Choi et al. [14]. Mitsik et al. [13] proposed clothing that provides cooling of the body based on a system of Peltier elements. Their design provides cooling of the upper body by 5 K. The authors stated that there is a non-linear dependence of the heat loss from the cooled body and the power generated by Peltier elements due to the thermal resistance of the electric circuit, which is also in accordance with the results obtained from our tests. However, we also proved that properly selected operation mode enables effective cooling by the designed system. In the case of Choi et al. [14], a similar effect was achieved with a use of a flexible heat sink. However, in this case other materials were used in order to achieve the flexibility of the designed solution. Together with our work, this proves that clothing ensuring thermoelectric cooling is a promising research direction with a high potential of implementation in the market thanks to high cooling capabilities and possible ergonomic clothing design due to its flexibility.

It should be also noted that our tests indicated the cooling effect is significantly influenced by heat sinks, which markedly lower the temperature of the skin under the modules before active cooling begins, i.e., Peltier modules are supplied. Despite the cooling effect achieved at the beginning of each test, the skin temperature under the modules at the end of the test in some cases was only slightly lower than the initial temperature. This is tantamount to a short time of effective operation of the cooling system. This is probably due to the declining efficiency of the heat sinks over time, resulting in heat accumulation on the hot side of the modules. This causes the temperature difference between the cold and warm sides of the modules to decrease, thereby reducing their cooling efficiency. Research in this topic will be continued using a selection of other fabrics with lower thermal resistance in order to provide the highest possible conduction from the hot side of the module, as well as lower water vapor resistance in order to provide the highest possible evaporation from the heat sink to the environment. Moreover, the efficiency of the developed cooling system will be also evaluated in other ambient temperatures. In future research, a prototype modified on the basis of the conclusions from these tests will be developed and evaluated in further laboratory tests with the participation of a group of end-users.

## 5. Conclusions

Laboratory tests of thermoelectric modules system with human participation allowed the most favorable control mode and it its parameters to be determined in terms of cooling efficiency. In the control mode ultimately selected, there was 1 min of cooling with the use of automatic mode with the cooling power limited to 2 W, followed by a 1-min break in the operation of the modules, during which the cooling power was 1 W. The proposed control mode allows the user to avoid a sudden increase in skin temperature under the cells during the standby mode of the modules due to the limitation of cooling power. It must be noted that the complex character of the most efficient control mode results from the type of the heat sinks applied, the operation of which mainly relies on heat accumulation by an absorbed fluid.

Based on the study, the optimal arrangement of the Peltier modules on the clothing was also selected, with two modules on the abdomen, two modules on the shoulder blades, and three modules on the lower back.

The dedicated electronic controlled system performed its task correctly. It was able to regulate the Peltier module supply power at the value set either by the user (manual mode) or by the higher-level temperature controller (automatic mode). A simple proportional controller with feedforward is sufficient for this purpose. The PD temperature controller regulated the skin temperature as measured by the sensors as long as the total heat (pumped from the skin and dissipated by the Peltier modules) remained within the heat removal capability of the heat sinks.

## Figures and Tables

**Table 1:** Study stages with related Peltier module location arrangements and experimental conditions.

Stage Designation and Aim	Study No.	Arrangement of Peltier Module Locations	Control Mode ^1^	Ambient Temperature (°C)
Stage I— Selecting the control mode	1	2 modules on the chest +5 modules on the back	Automatic	30
	2	2 modules on the chest +5 modules on the back	Manual	30
	3	2 modules on the chest +5 modules on the back	Mode 10/5//0.5— 10 min/5 min (auto/0.5 W)	30
	4	2 modules on the chest +5 modules on the back	Mode 1/1//0.5— 1 min/1 min (auto/0.5 W)	30
	5	2 modules on the chest +5 modules on the back	Mode 1/1//1— 1 min/1 min (auto/1 W)	30
	6	2 modules on the chest +5 modules on the back	Mode 0.5/0.5//1— 0.5 min/0.5 min (auto/1 W)	30
	7	2 modules on the chest +5 modules on the back	Mode limited 1/1//1— 1 min/1 min (auto/1 W), max power 2 W	30
Stage II— Peltier module Arrangement selection	8	Arrangement I— 2 modules on the chest +5 modules on the back	Selected control mode as selected after Stage I	30
	9	Arrangement II— 2 modules on the abdomen +5 modules on the back	Selected control mode as selected after Stage I	30
	10	Arrangement III— 2 modules on the abdomen +2 modules on the chest +3 modules on the back	Selected control mode as selected after Stage I	30

^1^ Where the control mode is labelled as “Mode X/Y//Z”, this refers to X minutes of cooling with the use of automatic mode, followed by Y minutes of break in modules’ operation, during which break the cooling power was Z watts.

## Data Availability

The data presented in this study are available on request from the corresponding author.
